# miR-140-5p induces cell apoptosis and decreases Warburg effect in chronic myeloid leukemia by targeting SIX1

**DOI:** 10.1042/BSR20190150

**Published:** 2019-04-30

**Authors:** Zi-Yuan Nie, Xiao-Jun Liu, Ying Zhan, Meng-Han Liu, Xiao-Yan Zhang, Zi-Ye Li, Ya-Qiong Lu, Jian-Min Luo, Lin Yang

**Affiliations:** Department of Hematology, The Second Hospital of Hebei Medical University, 215 Heping W Rd, Shijiazhuang 050000, China

**Keywords:** apoptosis, Chronic myeloid leukemia, miR-140-5p, PKM2, SIX1, Warburg effect

## Abstract

microRNAs (miRNA), as tumor suppressors or oncogenes, are involved in modulating cancer cell behavior, including cell proliferation and apoptosis. The miR-140-5p acts as a tumor suppressor in several tumors, but the role of miR-140-5p in chronic myeloid leukemia (CML) remains unclear. Here, we investigated the suppression of miR-140-5p in CML patients and CML cell lines using quantitative PCR (qPCR) and fluorescence *in situ* hybridization (FISH). Overexpression miR-140-5p in CML cells significantly inhibited cell proliferation as revealed by the CCK-8 assay and promoted cell apoptosis as revealed by flow cytometry. Moreover, the sine oculis homeobox 1 (SIX1) gene had been confirmed as a direct target of miR-140-5p using bioinformatics analysis and luciferase reporter assays. Overexpression of miR-140-5p decreased the SIX1 protein level in CML cells. SIX1 mRNA and protein levels were significantly up-regulated in CML patients and CML cell lines. Knockdown of SIX1 expression significantly inhibited CML cell proliferation and promoted cell apoptosis. Furthermore, SIX1 as a transcriptional factor positively regulated pyruvate kinase isozyme type M2 (PKM2) expression and played an important role in the Warburg effect. In addition, these findings indicated that miR-140-5p functions as a tumor suppressor and plays a critical role in CML cell apoptosis and metabolism by targeting SIX1. Moreover, the miR-140-5p/SIX1 axis may be a potential therapeutic target in CML.

## Introduction

Chronic myeloid leukemia (CML) is a myeloproliferative neoplasm characterized by fusion of the Abelson murine leukemia (ABL) gene on chromosome 9 with the breakpoint cluster region (BCR) gene on chromosome 22, which results in the expression of an oncoprotein, termed BCR-ABL1 tyrosine kinase [1–3]. Although tyrosine kinase inhibitors (TKIs) have been found to dramatically alter the natural progression of CML, improving 10-year overall survival (OS) from approximately 20% to 80–90%, chemotherapy resistance and intolerance are the main focus during the therapy of CML [4]. In addition, side-effects, such as skin toxicity and allergic reaction, remain serious clinical problems. Therefore, a better understanding of CML in terms of molecular mechanisms and alternative therapeutic avenues are urgently needed.

miRNAs are small non-coding RNAs which negatively regulate gene expression by targeting mRNAs at the UTR. It has previously been demonstrated that miRNAs serve important roles in numerous biological processes, including cell growth, cell cycle progression, apoptosis, migration, and invasion [5]. Dysregulated miRNAs may act as either oncogenes or tumor suppressors, depending on the biological function of their targets [6]. Accumulating evidence has suggested that miRNAs play an important role in CML development and progression. Certain miRNAs, such as miR-362-5p [7], miR-320a [8], miR-15-5p [9] and miR-486 [10], have been confirmed to act as oncogenes or tumor suppressor genes in CML. There are several other miRNAs speculated to be dysregulated, for which the molecular mechanisms in CML are still not well elucidated. miR-140-5p has been characterized as a tumor suppressor in gastric cancer (GC) [11], hepatocellular cancer [12], and hypopharyngeal cancer. Despite a strong antitumor effect of miR-140-5p in multiple types of cancers is associated with dysregulation of its target mRNAs. However, the role of miR-140-5p in CML has not been investigated yet.

Transcription factor sine oculis homeobox 1 (SIX1) is a developmentally regulated homeoprotein that plays a crucial role in the development of various organs and is not expressed in most normal adult tissues [13]. SIX1 is re-expressed in many cancers including breast [14], ovarian [15], colorectal [16], and hepatocellular carcinoma. Importantly, SIX1 regulates expression of many glycolytic genes, such as pyruvate kinase isozyme type M2 (PKM2), which modulate cell proliferation and/or apoptosis. In addition, SIX1 is a key transcription factor involved in the Warburg effect by regulating PKM2 expression [17]. However, the expression of SIX1 in CML and underlying metastasis has not yet been studied.

In the present study, we detected down-regulation of miR-140-5p in CML peripheral blood mononuclear cells (PBMCs). Furthermore, overexpression of miR-140-5p induced apoptosis and suppressed the growth of CML cells. As the direct target gene of miR-140-5p, SIX1 is up-regulated in CML PBMCs and down-regulated SIX1 level promotes CML cell apoptosis. In addition, SIX1, as a transcriptional factor, regulated PKM2 expression. miR-140-5p/SIX1 axis acts as a key regulator of the Warburg effect and cell survival in CML patients.

## Materials and methods

### Patients and specimens

Specimen collection Peripheral blood samples were collected from 30 CML patients, who were admitted to the Department of Hematology of the Second Hospital of Hebei Medical University between May 2016 and June 2017. Peripheral blood samples from 30 healthy donors were selected to serve as controls. The characteristics of CML patients and healthy donors are summarized in Table 1. PBMCs were isolated via lymphocyte separation. The inclusion criteria were (1) diagnosis of CML-CP via bone marrow morphology, immunology, molecular biology, and cytogeneticity; (2) clear pathological staging; (3) no chemotherapy was administered before the specimens were collected; and (4) availability of intact clinical data. The exclusion criteria were (5) significant organ dysfunction; (6) pregnancy (in females); and (7) failure to provide informed consent. The study protocol was approved by the Ethics Committee of Second Hospital of Hebei Medical University and written consent was obtained from each patient and healthy donor. All of experiments in this paper obey World Medical Association Declaration of Helsinki.

**Table 1:** Characteristics

Characteristics	CML-CP (*n*=30)	healthy donors (*n*=30)
Age (years), median (range)	42 (29–71)	42 (29–71)
Male/Female, (n/n)	18/12	18/12
WBCs, ×10^9^/l, median (range)	249 (39–405)	6.1 (4.9–9.3)
Hb level (g/l)	99 (81–146)	139 (121–155)
PLT count, ×10^9^/l, median (range)	464 (94–797)	221 (104–312)

Hb, hemoglobin; PLT, platelet; WBC, white blood cell.

### Cell culture and transfection

CML cell lines (KCL22 and K562) were maintained in our laboratory. KCL22 cells were cultured in Iscove’s modified Dulbecco’s medium (IMDM; Gibco, Beijing, China), which contained 10% fetal bovine serum (FBS) (Clark Bio, Claymont, DE, U.S.A.), 100 units/ml penicillin, and 100 μg/ml streptomycin. K562 cells were cultured in RPMI 1640 medium (Gibco, Beijing, China) supplemented with 10% FBS and the two antibiotics listed above. All kinds of cells were incubated at 37°C in a humidified incubator with 5% CO_2_. According to the manufacturer’s protocol, the transfection of all cells was carried out using Lipofectamine 2000 (Invitrogen). In brief, 2 × 10^6^ K562 or KCL22 cells were suspended in the 5 ml serum-free medium then transfected with 600 pmol siRNA with 10 μl lipo-2000 for 48 h. The miR-140-5p mimics, mimic-NC, miR-140-5p inhibitor, si-SIX1, and si-NC were purchased from GenePharma Co., Ltd (Shanghai, China). The sequence of si-SIX1 as follows: siSIX1-F:GGUGGACUUUCACAAAUAUTT; siSIX1-R:AUAUUUGUGAAAGUCCACCTT. Plasmid overexpression of SIX1 was purchased from GENEWIZ Company (Suzhou, China). After 24 or 48 h of transfection, the cells were harvested and lysed for western blotting, and the total RNA was extracted for quantitative real-time PCR (qRT-PCR).

### CCK-8 assays

After transfection was conducted, CCK-8 assays were performed for 24, 48, or 72 h in accordance with the manufacturer’s manual. Briefly, KCL22 and K562 cells were seeded into 96-well plates (2 × 10^4^ cells/well). We cultured them for 0, 24, 48, and 72 using IMDM medium or PRMI1640 medium at 37°C. The proliferation of KCL22 and K562 cells was determined by using an CCK-8 assays. After cultivation, 10 μl CCK-8 (Beibo, Beijing, China) was added to each well and the 96-well plates were incubated at 37°C in a humidified 5% CO_2_ atmosphere for 2.5 h. Absorbance was then read at 450 nm, in a microplate reader (Thermo Fisher, U.S.A.).

### Apoptosis assays

KCL22 and K562 cells were seeded into 6-well plates after transfection and treated according to the manufacturer’s instructions. The cells were stained with 5 μl of AnnexinV-FITC and 5 μl of PI (BD Bioscience Pharmingen, U.S.A.) and then analyzed with a BD FACSCanto II system (BD, U.S.A.). Briefly, turn on the 488-nm laser on the flow cytometer, and set up dot plots to detect FITC (520 nm) and PI (620 nm). Next, run an untreated sample and set the voltage and gain for FSC and SSC to ensure your live cells can be detected. Then, run a treated sample stained with FITC alone or PI alone to adjust the voltage and gain for the FITC detector and PI detector. Last, run a treated sample stained with FITC and PI. Adjust the compensation so that the live cells appear in the bottom left, the apoptotic cells appear in the bottom right, and the necrotic cells appear in the top right quadrants. Run each sample and acquire data for 20,000 events. The apoptosis was performed using BDFAC-Diva (BD, U.S.A.).

### RNA extraction and qRT-PCR

PBMCs and cultured cells were lysed using QIAzol Lysis Reagent (QIAGEN, 79306, Germany). The concentration and purity of the RNA were determined by using NanoDrop 2000 (Thermo Fisher). For microRNA, the miScripIIRT kit (QIAGEN GmbH, D-40724 Hilden, Germany) was used for reverse transcription, and the miScript SYBR® Green PCR kit was used for qRT-PCR according to the manufacturer’s protocol with following primers: miR-140-5p: GGCAGTGGTTTTACCCTATGGTAG; RNU6b (U6) AAAATATGGAACGCTTCACGAATT TGC. For large mRNA analysis, reverse transcription of RNA was performed by using the M-MLV First Strand Kit (Life Technologies). The Platinum SYBR Green quantitative PCR (qPCR) Super Mix UDG Kit (Invitrogen) was used for the qRT-PCR of mRNAs. The real-time PCR experiments were carried on a CFX96™ Real-Time System (Bio-Rad) with primers of SIX1-F:CCAGGTCAGCAACTGGTTTAAG, SIX1-R:ATAGTTTGAGCTCCTGGCGT; PKM2-F:GTCTGGGAGGAAAGTCGCTC, PKM2-R:ACGCTGCAAAGACGAAGAGA. All data were normalized with β-actin and analyzed by adopting 2^−ΔΔC_t_^ method.

### Western blot analysis

Western blotting was performed as described previously [28]. In brief, frozen tissue samples were homogenized in RIPA lysis buffer (50 mM Tris-HCl, pH 7.5, 150 mM NaCl, 1% NP-40, 0.5% Na-deoxycholate, and 0.1% SDS), and cultured cells were lysed with lysis buffer (1% Triton X-100, 150 mM NaCl, 10 mM Tris-HCl, pH 7.4, 1 mM EDTA, 1 mM EGTA, pH 8.0, 0.2 mM Na3VO4, 0.2 mM phenylmethylsulfonyl fluoride, and 0.5% NP-40). Equal amounts of protein were run on 10% SDS–PAGE, and electro-transferred to a polyvinylidene fluoride (PVDF) membranes (Millipore) with primary antibodies overnight at 4°C. The antibodies that were used were as follows: anti-SIX1 (1:500, ab211359), anti-PKM2 (1:1000, ab137852), anti-apoptosis regulator Bcl-2 (anti-BCL-2) (1:1000, 12789-1-AP), anti-BAX (1:1000, 50599-2-Ig), or anti-β-actin (1:1000, sc-47778). Membranes were then incubated with the secondary antibody (1:5000, Rockland) for 1 h at room temperature. The blots were treated with the Immobilo™ Western (Millipore), and detected by ECL (enhanced chemiluminescence) Fuazon Fx (Vilber Lourmat). Images were captured and processed by FusionCapt Advance Fx5 software (Vilber Lourmat). All experiments were replicated three times.

### *In situ* hybridization

*In situ* hybridization was performed as described previously [18]. In brief, according to user manual of miRCURY LNATM microRNA ISH Optimization Kit (Exiqon), cultured cells smears were deparaffinized and rehydrated for fluorescence *in situ* hybridization (FISH). Hybridization was performed using fluorescence-labeled miR-140-5p probes with hybridization buffer (Exiqon) by incubation at 56°C for 1 h in a thermo-block (Labnet, U.S.A.). After stringent washing with SSC buffer and PBS, images were acquired by using a Leica microscope (Leica DM6000B, Switzerland) and digitized with a software of LAS V.4.4 (Leica). miR-140-5p probe was purchased from GenePharma Co., Ltd (Shanghai, China). The sequence of miR-140-5p-cy3-probe was as follows: TACCATAGGGTAAAACCACTG. The *in situ* hybridization image analysis was done according to the protocol of FISH image analysis in clinical diagnosis. Briefly, 200 cell nuclei were (stained with DAPI) counted under fluorescence microscopy. The FISH-positive cells were stained with red probes and emitted red fluorescence. Positive-cell numbers (per 200) cells were used to statistical analysis. Each experiment was repeated three times.

### Vector construction and luciferase reporter assay

All plasmids were constructed using restriction-enzyme digestion and one-step cloning (ClonExpress II One Step Cloning Kit, C112-02; Vazyme Biotech Co., Ltd., Nanjing, P.R. China) or recombinant methods. The UTR sequences of SIX1 containing WT or mutant (mut) forms of the miR-140-5p target site were inserted into the *Xho1* and *Sal1* digested-pmir-GLO Dual-Luciferase miRNA Target Expression Vector (Promega Corp., Madison, WI, U.S.A.). A 2 kb PKM2 promoter sequence was obtained by PCR with primer and inserted into the *Mlu1* and *Xho1* digested-pGL3-basic vector (Promega Corp., Madison, WI, U.S.A.). Luciferase assay was performed as described previously [18]. In brief, K562 cells were seeded into a 24-well plate, PKM2 reporter construct or the empty reporter vector was co-transfected with pcDNA3.1-SIX1 and pRL-TK, or co-transfected with pcDNA3.1-NC and pRL-TK. After 24 h of transfection, luciferase activity was measured using a Dual-Glo Luciferase Assay System (Promega, Madison, WI) with a Flash and Glow (LB955, Berthold Technologies) reader. The specific target activity was expressed as the relative activity ratio of firefly luciferase to Renilla luciferase.

### Chromatin immunoprecipitation (ChIP) assay

The chromatin immunoprecipitation (ChIP) assay was performed as described previously [19]. According to the manufacturer’s protocol of EZ-CHIP™ Chromatin Immunoprecipitation Kit (Millipore, #17–371), cells were crosslinked with 1% formaldehyde and sonicated to an average size of 400–600 bp. SIX1 antibody and normal mouse IgG control were used for ChIP, respectively. The precipitated DNA was purified and analyzed by qRT-PCR amplification using primers of SIX1-PKM2-chip-F:CGGGGCCGGGAGAATGCTG, SIX1-PKM2-chip-R1:CCTGATGACCAATGGGGACGC specific for the PKM2 promoter.

### Target prediction

Potential target genes of SIX1 were identified with following miRNA target prediction algorithms: miRanda (www.microrna.org) and Targetscan (http://www.targetscan.org) [19]. The inclusion criteria of target gene were: (1) appeared in both databases; (2) scores in Targetscan >95.

### Statistical analysis

All of the data were represented as the means ± S.E.M. ANOVA and paired two-tailed Student’s *t* tests were used to analyze the statistical significance of differences of data. Results were considered statistically significant at *P* < 0.05. Statistical analysis was performed using Graphpad Prism 7.0 software (GraphPad Software, San Diego, CA, U.S.A.).

## Results

### miR-140-5p is down-regulated in CML patients and CML cell lines

**Figure 1:**
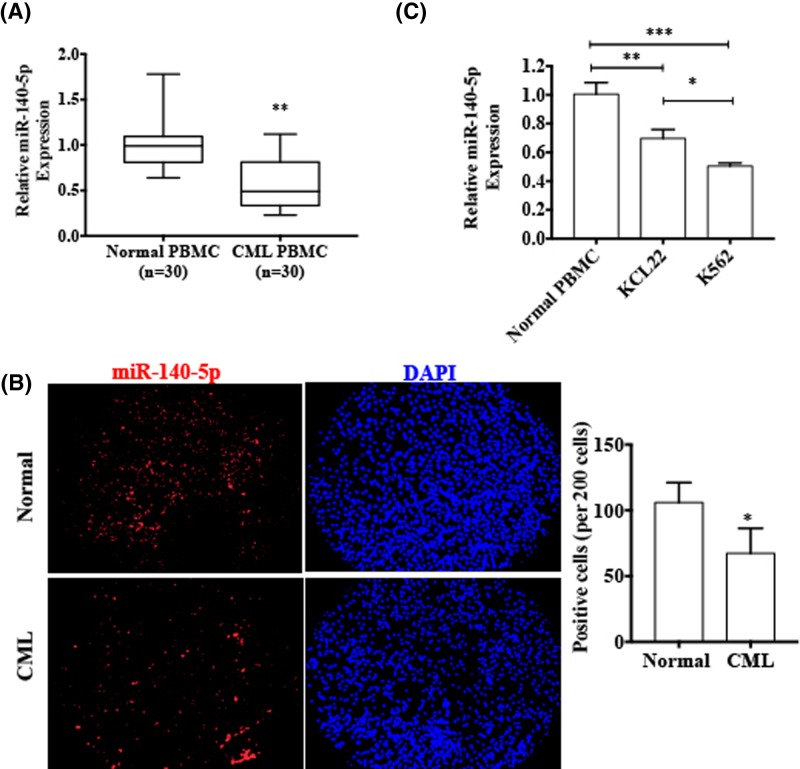
miR-140-5p is down-regulated in CML patients and CML cell lines (**A**) qRT-PCR was used to detect and compare the miR-140-5p expression in CML patients’ PBMCs with healthy donors’ PBMCs. ^*^P < 0.001 vs normal PBMCs. Normalized against an internal control U6 RNA**.** (**B**) FISH for detection of miR-140-5p in CML patients’ PBMCs and healthy donors’ PBMCs. Blue staining represents the nucleus and red staining indicates miR-140-5p. Scale bar  =  64 μm. Right panel shows the number of FISH-positive cells of three independent experiments (per 200 cells) *P < 0.001 vs normal PBMCs. (**C**) The expression levels of miR-140-5p were assessed in CML cell lines (K562 and KCL22) and healthy donors’ PBMCs. ^***^P < 0.001, ^**^P < 0.01 vs normal PBMCs.

To validate the expression of miR-140-5p in CML patients, we used qRT-PCR to compare the miR-140-5p expression in the PBMCs of 30 CML patients and PBMCs of normal individuals. As shown in Figure 1A, CML patients’ PBMCs expressed significantly lower levels of miR-140-5p compared with normal person’s PBMC (*P*<0.001). Further, RNA *in situ* hybridization also showed that miR-140-5p was markedly down-regulated in CML patients’ PBMCs (Figure 1B). In addition, we also examined miR-140-5p expression in the different CML cell lines (K562 and KCL22), with healthy donors’ PBMCs as control, using qRT-PCR. The results showed that miR-140-5p expression was down-regulated in both CML cell lines (Figure 1C). These findings revealed that the miR-140-5p was down-regulated in CML.

### Overexpression of miR-140-5p promotes CML cell apoptosis *in vitro*

**Figure 2:**
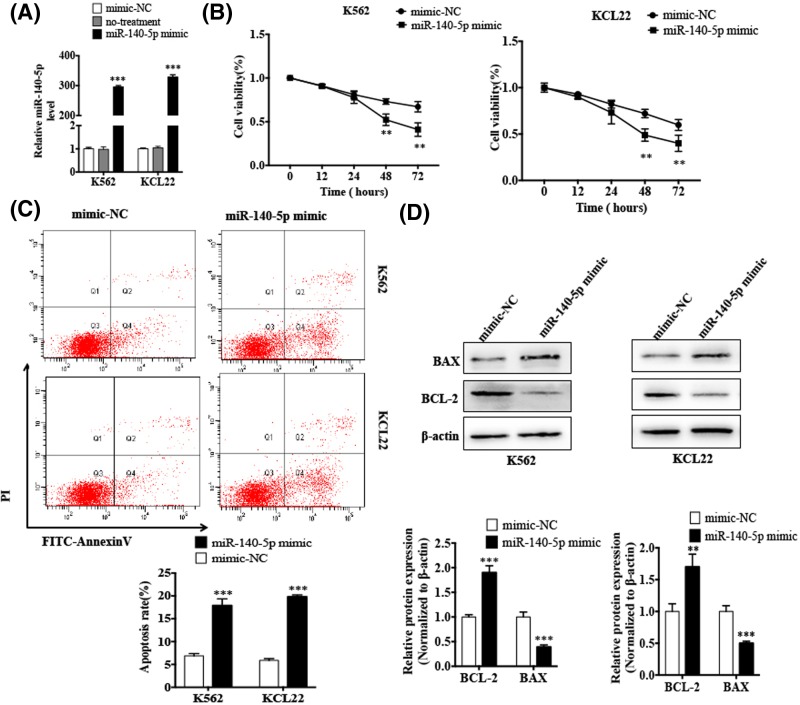
Overexpression of miR-140-5p promotes CML cell apoptosis *in vitro* (**A**) K562 and KCL22 cells were transfected with the miR-140-5p mimic or mimic-NC for 48 h, followed by qRT-PCR to assess miR-140-5p expression in both cell lines. ^***^P < 0.001 vs mimic-NC. (**B**) K562 and KCL22 cells were transfected with either the miR-140-5p mimic or mimic-NC for 12, 24, 48 and 72 h, and CCK-8 assay was performed to detect the cell viability. ^**^P < 0.01 vs mimic-NC. (**C**) K562 and KCL22 cells were treated as in (A), flow cytometry detected cell apoptosis. Right panel shows the apoptosis rate of three independent experiments.^**^P< 0.001 vs mimic-NC. (**D**) K562 and KCL22 cells were treated as in (A), and western blotting was done to detect expression levels of BAX and BCL-2 proteins. β-actin was used as an internal control. Bottom panel shows densitometric analysis of three independent experiments. ^**^P<0.01, ^***^P<0.001 vs mimic-NC.

Since we found that miR-140-5p was down-regulated in both CML patients’ PBMC and CML cell lines, we investigated whether miR-140-5p played a role in CML cells. First, K562 and KCL22 cells were transfected with miR-140-5p mimic or mimic-NC, transfection efficiency was detected by qRT-PCR. As shown in Figure 2A, miR-140-5p mimic dramatically increased the miR-140-5p expression. Then, we determined the cell viability by the CCK-8 assay. As shown in Figure 2B, cell viability of K562 and KCL22 cells were significantly affected 48 h after transfection with miR-140-5p mimic. Furthermore, we used flow cytometry analysis to investigate whether overexpression of miR-140-5p affected cell apoptosis. As shown in Figure 2C, overexpression of miR-140-5p induced apoptosis in K562 and KCL22 cells. In addition, we determined the expression levels of apoptosis-related genes, BCl-2 and BAX by western blotting. The results showed that the overexpression of miR-140-5p enhanced the BAX protein expression, whereas decreased the BCL-2 protein expression in both K562 and KCL22 cells (Figure 2D). These results demonstrated that overexpression of miR-140-5p promoted apoptosis in CML cells.

### SIX1 is a direct target of miR-140-5p

**Figure 3:**
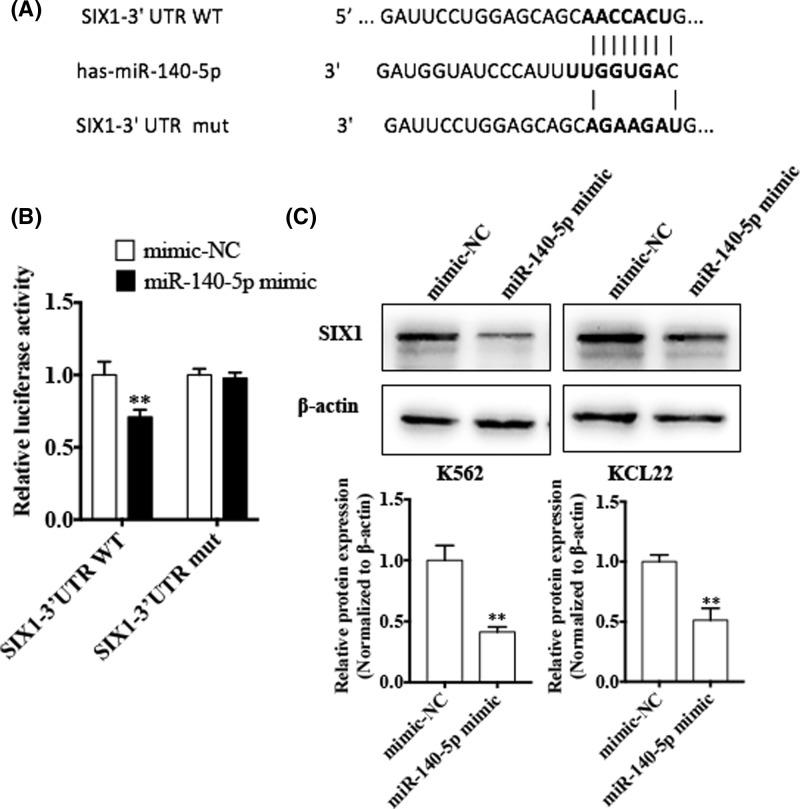
SIX1 is a direct target of miR-140-5p (**A**) Prediction of miR-140-5p binding site in SIX1 3′-UTR. (**B**) Luciferase reporter assays were performed in K562 cells co-transfected with the miR-140-5p mimic and either the WT or mut SIX1 3′-UTR-luciferase reporter. ^**^P<0.01 vs mimic-NC. (**C**) K562 and KCL22 cells were transfected with the miR-140-5p mimic and mimic-NC. Western blotting was done to analyze the SIX1 protein expression. β-actin was used as an internal control. Bottom panel shows densitometric analysis of three independent experiments. ^**^P<0.01 vs mimic-NC.

To identify potential target gene regulated by miR-140-5p, we used two target prediction programs, miRanda and TargetScan. We found that the SIX1 3′-UTR contains a putative miR-140-5p binding site (Figure 3A). Subsequently, to confirm whether SIX1 is a direct target of miR-140-5p, we co-transfected K562 cells with the miR-140-5p mimic and either the WT or mut SIX1 3′-UTR-luciferase reporter, and observed that the miR-140-5p mimic significantly decreased luciferase activity mediated by wild-type SIX1 3′-UTR; mutation of the miR-140-5p-binding site in SIX1 almost completely restored the luciferase activity in the presence of the miR-140-5p mimic (Figure 3B). Further, K562 and KCL22 cells were transfected with the miR-140-5p mimic and SIX1 expression was detected by western blotting. The results showed that the miR-140-5p mimic significantly reduced the protein levels of SIX1 (Figure 3C). Taken together, these data suggested that miR-140-5p down-regulated SIX1 expression by directly targeting the miR-140-5p-binding site in the SIX1 3′-UTR.

### SIX1 is up-regulated in CML patients and CML cell lines

**Figure 4:**
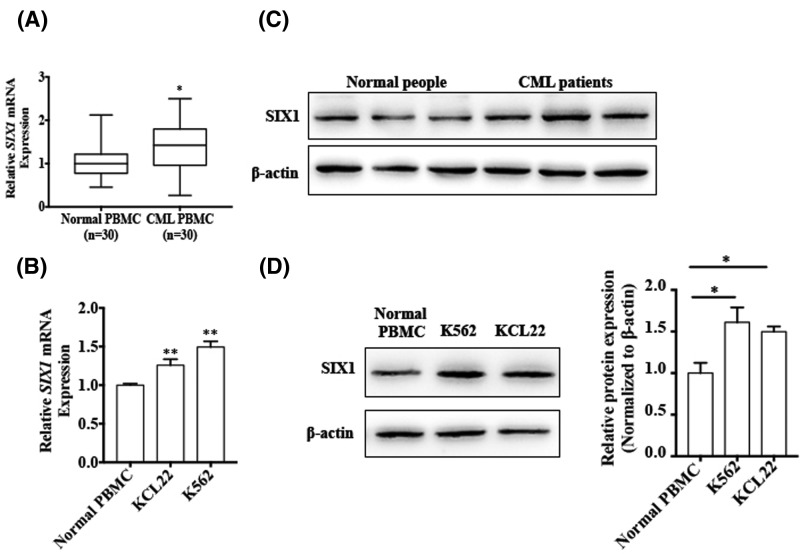
SIX1 is up-regulated in CML patients and CML cell lines (**A**) qRT-PCR was used to detect the SIX1 mRNA expression in CML patients’ PBMCs compared with normal individuals’ PBMCs. ^*^P < 0.05 vs normal individuals’ PBMCs. Normalized against an internal control, β-actin**.** (**B**) qRT-PCR was used to detect the SIX1 mRNA expression in CML cell lines compared with normal individuals’ PBMCs. ^**^P < 0.01 vs normal individuals’ PBMCs. Normalized against an internal control, β-actin**.** (**C**) Western blotting was done to analyze the SIX1 protein level in CML patients’ PBMCs and normal individuals’ PBMCs. (**D**) The protein levels of SIX1 were detected in K562 and KCL22 cells using Western blotting, compared with normal individuals’ PBMCs. β-actin was used as an internal control. Right panel shows densitometric analysis of three independent experiments. *P<0.05 vs normal PBMCs.

Although SIX1 is a known oncogene overexpressed in many cancers, its expression in CML is unknown. Thus, we sought to investigate the expression of SIX1 in CML patients’ PBMCs and CML cell lines. First, we used qRT-PCR to detect SIX1 mRNA expression in CML patients’ PBMCs and normal individuals’ PBMCs. As shown in Figure 4A, SIX1 mRNA level was higher in CML patients’ PBMCs compared with those from normal individuals’ PBMCs. We also examined SIX1 mRNA expression in the different CML cell lines and normal individuals’ PBMCs. The result showed that miR-140-5p expression was significantly increased in both CML cell lines (Figure 4B). In addition, we used western blotting to detect the SIX1 protein levels in CML patients and CML cell lines. As shown in Figure 4C,D, SIX1 protein expression is increased in CML patients and CML cell lines. These results suggested that SIX1 might act as an oncogene in CML.

### miR-140-5p induces CML cell apoptosis by suppressing SIX1

**Figure 5:**
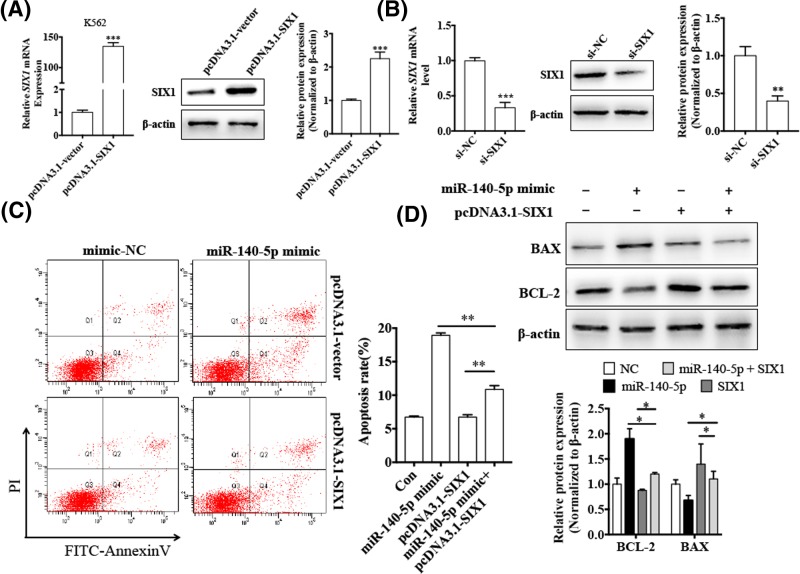
miR-140-5p promotes apoptosis of CML cells through down-regulation of SIX1 expression (**A**) K562 cells were transfected with pcDNA3.1-SIX1 or negative control pcDNA3.1-vector. SIX1 mRNA and protein levels were detected by qRT-PCR and western blotting, respectively. β-actin was used as an internal control. Right panel shows densitometric analysis of three independent experiments.^***^P < 0.001 vs pcDNA3.1-vector. (**B**) K562 cells were transfected with si-SIX1 or si-NC. SIX1 mRNA and protein levels were detected by qRT-PCR and western blotting, respectively. β-actin was used as an internal control. Right panel shows densitometric analysis of three independent experiments.^**^P < 0.01, ^***^P < 0.001 vs si-NC. (**C**) K562 cells were transfected with the miR-140-5p mimic, pcDNA3.1-SIX1, or co-transfected with both miR-140-5p and pcDNA3.1-SIX1, and cell apoptosis was analyzed by flow cytometry. Right panel shows the apoptosis rate of three independent experiments.^**^P < 0.01 vs corresponding control. (**D**) K562 cells were treated as (C), and levels of BCL-2 and BAX proteins were detected using western blotting. β-actin was used as an internal control. Bottom panel shows densitometric analysis of three independent experiments. ^*^P < 0.05 vs corresponding control.

Since, miR-140-5p was down-regulated and SIX1 was up-regulated in CML cells, and overexpression of miR-140-5p promoted CML cell apoptosis, we investigated whether miR-140-5p down-regulated SIX1, leading to cell apoptosis. First, we transfected K562 cells with pcDNA3.1-SIX1 to overexpress SIX1. As shown in Figure 5A,B, both mRNA and protein levels of SIX1 were significantly increased compared with the negative control. Furthermore, we examined whether overexpression of SIX1 influences the effects of miR-140-5p on cell apoptosis. The result showed that, compared with K562 cells transfected with the miR-140-5p mimic alone, the apoptosis rate was decreased in K562 cells co-transfected with the miR-140-5p mimic and pcDNA3.1-SIX1; that is, overexpression of SIX1 decreased the apoptosis induced by miR-140-5p (Figure 5C). Consistently, western blot analysis showed that overexpression of miR-140-5p and SIX1 in K562 cells markedly suppressed BAX expression and enhanced BCL-2 expression compared with K562 cells transfected with the miR-140-5p mimic alone (Figure 5D). The above results suggested that SIX1 mediated cell apoptosis induced by miR-140-5p in CML cells.

### SIX1 decreases Warburg effect by regulation of PKM2 gene in CML cells

**Figure 6:**
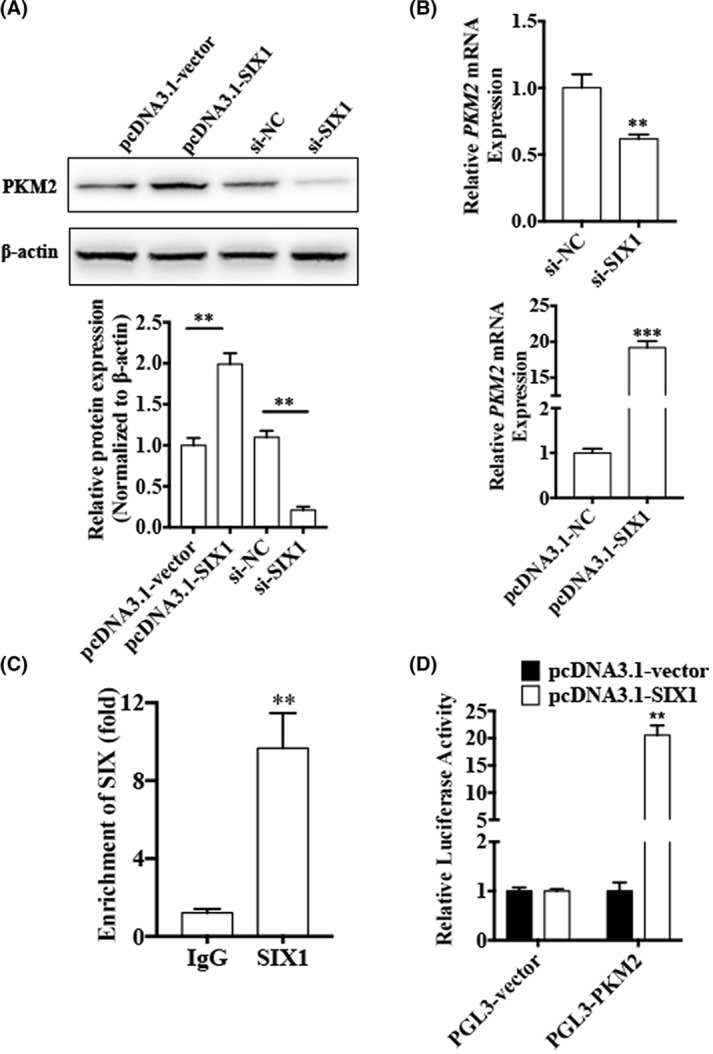
miR-140-5p induces CML cell apoptosis by suppressing SIX1 in CML cells (**A,B**) K562 cells were transfected with pcDNA3.1-SIX1, negative control pcDNA3.1-vector, si-SIX1, or si-NC. PKM2 mRNA and protein levels were detected using qRT-PCR and western blotting. β-actin was used as an internal control. Bottom panel shows densitometric analysis of three independent experiments. ^**^P < 0.01, ^***^P < 0.001 vs corresponding control. (**C**) ChIP-qPCR was used to detect binding of SIX1 to the PKM2 promoter region in K562 cells. Arrowheads indicate the position of primers used for ChIP-PCR. ^**^P<0.01 vs IgG. (**D**) K562 cells were co-transfected with PKM2 promoter reporter construct and either pcDNA3.1-SIX1 or pcDNA3.1-NC. Luciferase reporter assays were performed. ^*^P < 0.01 vs pcDNA3.1-vector.

Cancer cells exhibit aberrant metabolism characterized by high degree of glycolysis even in the presence of abundant oxygen. This phenomenon, known as the Warburg effect or aerobic glycolysis, facilitates tumor growth with elevated glucose uptake and lactate production [20]. PKM2, the final enzyme in glycolysis, is directly responsible for the Warburg effect [21]. A previous study has shown that SIX1 is a transcriptional activator that acts as a key regulator of the Warburg effect in HepG2 cells [17]. Hence, we investigated whether SIX1 regulated PKM2 expression in CML cells. As we speculated, SIX1 overexpression led to significantly increased mRNA and protein levels of PKM2 in K562 cells. Conversely, knockdown of SIX1 decreased mRNA and protein levels of PKM2 (Figure 6A,B). These results confirmed that PKM2 was positively regulated by SIX1 in K562 cells. Then, we used a luciferase reporter assay to detect whether SIX1 regulates PKM2 gene expression at transcriptional level. As shown in Figure 6C, overexpression of SIX1 significantly enhanced the PKM2 promoter-driven luciferase activity. To further examine the effects of SIX1 on PKM2 expression, ChIP-qPCR was performed. The results showed that endogenous SIX1 was recruited to the regions containing the specific binding sites in PKM2 promoter (Figure 6D). These data suggested that SIX1 promotes PKM2 gene transcription by binding to its promoter.

### The miR-140-5p/SIX1 axis regulates PKM2 expression in CML cells

**Figure 7:**
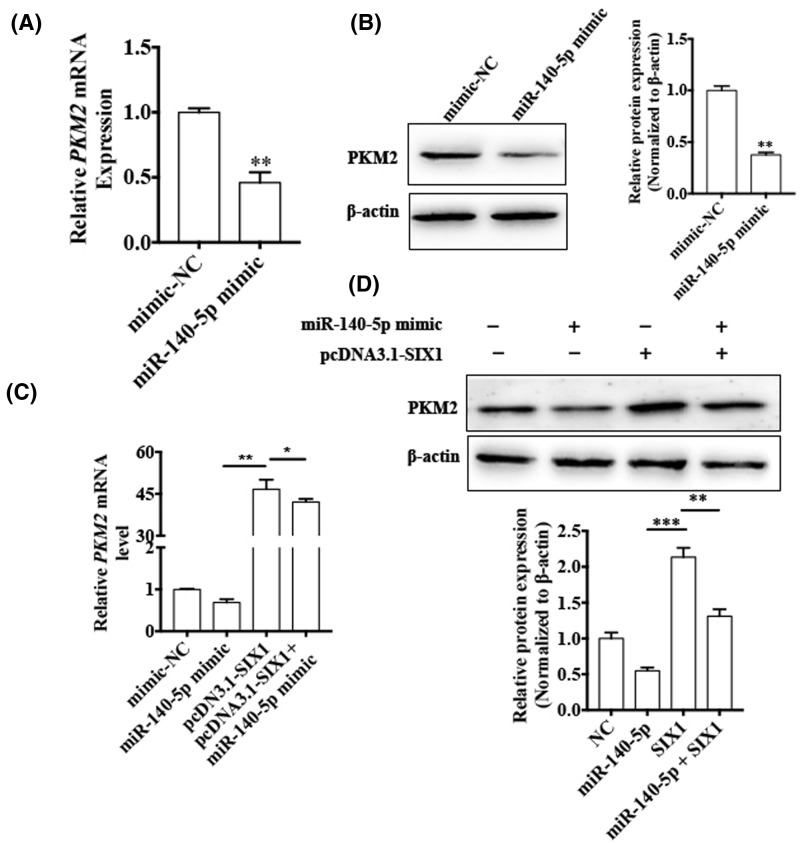
The miR-140-5p/SIX1 axis regulates PKM2 expression in CML cells (**A,B**) K562 cells were transfected with either the miR-140-5p mimic or mimic-NC. PKM2 mRNA and protein levels were detected using qRT-PCR and Western blotting, respectively. β-actin was used as an internal control. Right panel shows densitometric analysis of three independent experiments. ^**^P < 0.01 vs mimic-NC. (**C,D**) K562 cell were transfected with the miR-140-5p mimic, pcDNA3.1-SIX1, or co-transfected with both miR-140-5p and pcDNA3.1-SIX1. PKM2 mRNA and protein levels were detected by qRT-PCR and western blotting, respectively. β-actin was used as an internal control. Bottom panel shows densitometric analysis of three independent experiments. ^**^P < 0.01, ^***^P < 0.001 vs corresponding control.

Since SIX1 is a direct downstream target of miR-140-5p and it regulated PKM2 expression, we sought to investigate the relationship between miR-140-5p and PKM2. First, K562 cells were transfected with either the miR-140-5p mimic or its negative control. The results showed that miR-140-5p overexpression dramatically reduced the mRNA and protein levels of PKM2 (Figure 7A,B). However, simultaneously overexpressed miR-140-5p and SIX1 increased both mRNA and protein levels of PKM2. In parallel, inhibition of PKM2 level by overexpression of miR-140-5p was also reverted by SIX1 overexpression (Figure 7C,D). These results indicated that miR-140-5p negatively regulated PKM2 expression and that overexpression of SIX1 reversed the effects of miR-140-5p in CML cells.

## Discussion

Accumulated evidence has demonstrated that miRNAs play significant roles in CML development [22,23]. These miRNAs may act as tumor suppressors or oncogenes based on the target gene being regulated [24]. Therefore, identification of miRNAs and their target genes in CML has been critical for understanding their roles in tumorigenesis and tumor development. More importantly, miRNAs have been investigated as novel therapeutic targets. However, so far, there is a paucity of data on the expression, functions, and targets of miR-140-5p in CML. In the present study, our results showed that miR-140-5p expression was significantly down-regulated in CML. In addition, overexpression of miR-140-5p expression inhibited proliferation and promoted apoptosis in CML. Furthermore, SIX1 was validated as a direct target gene of miR-140-5p in CML. miR-140-5p also regulated the Warburg effect by targeting SIX1. These findings suggested that miR-140-5p played tumor suppressive roles in CML cell survival and metabolism.

Expression of miR-140-5p has been investigated in various types of cancers. For example, in GC, miR-140-5p expression was found to be significantly down-regulated in GC tissues and cell lines and correlated with the patients’ OS (OS rate) [25]. Yang *et al* found that miR-140-5p expression was significantly decreased in hepatocarcinoma (HCC) tissues and all of six liver cancer cell lines examined, and its expression levels correlated with overall and disease-free survival of HCC patients [12]. Li et al. [26] showed that miR-140-5p expression level was lower in esophageal cancer cell lines and it induced epithelial-to-mesenchymal transformation (EMT) and promoted cell invasion. In the present study, we showed that miR-140-5p expression was significantly down-regulated in CML. Moreover, overexpression of miR-140-5p promoted apoptosis and induced proliferation in CML cells *in vitro*. The miR-140-5p might act as a tumor suppressor in CML. Whether miR-140-5p expression correlated with CML patient’s survival needs to be investigated in future.

Identification of miR-140-5p target genes is important for understanding its role in tumorigenesis and tumor progression. Several target genes of miR-140-5p have been identified, including platelet-derived growth factor receptor A (PDGFRA) in ovarian cancer [27], insulin-like growth factor 2 mRNA-binding protein 1 (IGF2BP1) in cervical cancer [28], and YES proto-oncogene 1 (YES1) in gastric cancer [25]. In the present study, we observed that miR-140-5p was able to directly target the 3′-UTR of SIX1 and negatively regulated SIX1 protein expression. In addition, SIX1 knockdown enhanced the effects induced by miR-140-5p overexpression on proliferation and apoptosis of CML cells. Furthermore, rescue experiments indicated that enforced SIX1 expression abolished the tumor suppressive roles of miR-140-5p and its effects on growth and apoptosis of CML by directly targeting SIX1.

SIX1, a member of the homeobox gene superfamily, regulates the development of multiple organs by mediating cell cycle regulators and inhibition of apoptosis. Six1^−/−^ mouse embryos exhibit defects in the proliferation and survival of the precursor cells of multiple organs and die at birth. Further, mechanistic studies have indicated that SIX1, as an oncogene, is overexpressed in breast cancer [14], colorectal cancer [29], esophageal squamous cell carcinoma [30], and pancreatic cancer [31], and is associated with the development, progression, and prognosis of multiple tumors. In the present study, we confirmed that SIX1 expression was up-regulated at both mRNA and protein levels in CML patients compared with healthy donors. Its expression was higher in CML cell lines too. The present study has demonstrated that, due to the crucial roles of SIX1 in CML, it could be developed as a therapeutic target for CML patients.

More recently, SIX1 has been shown to act as a major transcription factor playing a causal role in glycolysis regulation and is involved in the transcriptional regulation of glucose metabolism [17]. The Warburg effect is critical for tumor development [32, 33]; it represents a pro-oncogenic metabolism switch such that cancer cells take up higher levels of glucose than normal tissue and favor incomplete oxidation of glucose even in the presence of abundant oxygen [32, 34]. More than ten genes-encoding glycolytic enzymes are directly responsible for the Warburg effect [35]. PKM2, a rate-limiting enzyme in glycolysis, is crucial for aerobic glycolysis and provides a growth advantage to tumors [21, 36]. PKM2 expression has been found to be up-regulated in several types of cancers, including CML [37], and it promotes the Warburg effect. The expression of PKM2 is regulated at multiple levels via DNA methylation [38], pre-mRNA splicing of PKM [39], PKM2-specific miRNAs [40, 41], and transcription factor [42]. In the present study, we found that SIX1 directly targeted PKM2 promoter, and thus, transcriptionally regulated the expression of PKM2 in CML cells. Therefore, miR-140-5p regulated PKM2 by directly targeting SIX1 gene. However, how PKM2 affects cell survival of CML cells remains unknown and warrants further investigation.

## Conclusion

The present study is the first to report that miR-140-5p is down-regulated in CML patients and cell lines; whereas, SIX1 is overexpressed. Both of them affect CML cells survival and glycolytic metabolism, making the miR-140-5p/SIX1 axis a possible target for CML therapy.

## Abbreviations

ABL: Abelson murine leukemia
BAX: bcl-2-associated X protein
BCL-2: apoptosis regulator Bcl-2
BCR: breakpoint cluster region
CCK: cell counting kit
ChIP: chromatin immunoprecipitation
CML: chronic myeloid leukemia
CP: chornic phase
FBS: fetal bovine serum
FISH: fluorescence *in situ* hybridization
FITC: fluorescein isothiocyanate
IGF2BP1: insulin-like growth factor 2 mRNA-binding protein 1
IMDM: Iscove’s modified Dulbecco’s medium
mut: mutant
NC: negative control
OS: overall survival
PI: propidium iodide
PBMCs: peripheral blood mononuclear cells
PDGFRA: platelet-derived growth factor receptor A
PKM2: pyruvate kinase isozyme type M2
qPCR: quantitative PCR
qRT-PCR: quantitative polymerase chain reaction
SIX1: sine oculis homeobox 1
TKI: tyrosine kinase inhibitor
YES1: YES proto-oncogene 1
